# Psychological Distress, Social Support, Coping Style, and Perceived Stress Among Medical Staff and Medical Students in the Early Stages of the COVID-19 Epidemic in China

**DOI:** 10.3389/fpsyt.2021.664808

**Published:** 2021-06-01

**Authors:** Zhe Li, Xin Yi, Mengting Zhong, Zhixiong Li, Weiyi Xiang, Shuang Wu, Zhenzhen Xiong

**Affiliations:** ^1^Mental Health Center, West China Hospital, Sichuan University, Chengdu, China; ^2^Sichuan Clinical Medical Research Center for Mental Disorders, Chengdu, China; ^3^School of Nursing, Chengdu University, Chengdu, China; ^4^School of Nursing, Chengdu Medical College, Chengdu, China; ^5^The Third Department of Clinical Psychology, Karamay Municipal People's Hospital, Karamay, China; ^6^The West China College of Medicine, Sichuan University, Chengdu, China

**Keywords:** COVID-19, psychological distress, social support, coping style, stress, medical staff, medical students

## Abstract

**Background:** The COVID-19 pandemic has had impact that may contribute to a rise in mental health problems. The present study was aimed to better understand psychological status among medical staff and medical students during the early epidemic and to explore the influence factors of psychological distress.

**Methods:** A cross-sectional survey was conducted online from February 2–14, 2020. We collected general information related to the COVID-19 outbreak. Respondents were assessed using the Kessler-6 Psychological Distress Scale (K6), Social Support Rating Scale (SSRS), Perceived Stress Scale (PSS) and Simplified Coping Style Questionnaire (SCSQ). Stepwise multiple linear regression was performed to identify factors influencing psychological distress.

**Results:** Five hundred and twenty-eight respondents returned valid questionnaires. Medical staff and Medical students scored averages of 6.77 ± 5.04, 15.48 ± 8.66 on the K6, 37.22 ± 11.39, 22.62 ± 11.25 on the SSRS and 18.52 ± 7.54, 28.49 ± 11.17 on the PSS, respectively. Most medical staff (279, 91.77%) and 148 medical students (66.07%) showed a positive coping style. Social support, perceived stress, hours spent watching epidemic-related information per day and frequency of epidemic-related dreams were identified as factors influencing psychological distress among medical staff and medical students. Coping style emerged as a determinant of psychological distress among medical staff.

**Conclusions:** In the early stages of the COVID-19 epidemic in China, medical staff and medical students were at moderate to high risk of psychological distress. Our results suggest that psychological interventions designed to strengthen social support, reduce perceived stress and adopt a positive coping style may be effective at improving the mental health of medical staff and medical students.

## Introduction

After being declared an international public health emergency and then an epidemic within <2 months (1, 2), the novel coronavirus disease (COVID-19) epidemic has caused worldwide panic as the numbers of patients, suspected cases and affected regions have increased. As of September 7, 2020, data from the World Health Organization continue to show strong increases in new COVID-19 cases and deaths during the previous week; however, no effective treatment or targeted vaccine is yet available (3).

Many countries have implemented strict control measures in an unprecedented effort to contain the epidemic. Schools and businesses have closed, people have isolated themselves, and personal protective equipment has become scarce, contributing to a global atmosphere of fear, anxiety and depression (4). Overwhelming, sensationalist media coverage has intensified the psychological impact on the public, and may be causing more serious consequences than COVID-19 itself (5). The National Health Commission in China has mandated mental health strategies for patients, medical workers, and people in medical isolation in order to combat the psychological impact of the epidemic (6).

Medical staff, as front-line warriors in epidemic control and prevention, are at high risk of being infected and are continuously exposed to the stresses of providing clinical care under resource-limited conditions. When a new infectious disease outbreak, medical personnel are often at the highest risk of exposure. In the early stages of the epidemic in China, more than 3,000 medical staff in Hubei Province were infected, 40% of which occurred in hospitals (7). Overwork and worry about being infected may increase the risk of psychological distress among medical staff. The prevalence of various negative conditions was higher among medical health workers than among non-medical health workers, including insomnia (38.4 vs. 30.5%), anxiety (13.0 vs. 8.5%), depression (12.2 vs. 9.5%), somatization (1.6 vs. 0.4%), and obsessive-compulsive symptoms (5.3 vs. 2.2%) (8).

Medical students are an important force in the fight against the epidemic in the future, so their mental state when dealing with the epidemic also deserves attention. Studies have confirmed that medical students, in particular because of their professional background, pay close attention to the epidemic, leading them to experience excessive stress and concern (9). For example, in a study at Changzhi Medical College in China, 0.9% of students reported severe anxiety; 2.7%, moderate anxiety; and 21.3%, mild anxiety (9). Studies conducted during epidemics of Severe Acute Respiratory Syndrome (SARS), Middle East Respiratory Syndrome (MERS), and Ebola also identified varying degrees of psychological problems among medical staff and students (10–12). Although medical students have some medical training, it is still difficult and stressful for them to make decisions during epidemics due to their lack of clinical experience, particularly during emergency situations (13–15). Therefore, investigating their psychological status during an epidemic may help us better understand and train medical students in the future.

When faced with emerging outbreaks of infectious disease or traumatic experiences, people may respond differently according to their coping style, level of social support or perceived level of stress. This can lead to stronger or weaker psychological distress. Coping strategies refer to the specific efforts, both behavioral and psychological, that people employ to master, tolerate, reduce, or minimize stressful events (16). Coping styles in a disease outbreak are significantly correlated with mental state: positive coping can generate positive emotions and behaviors that lead to improved outcomes, while negative coping styles may be associated with serious psychological distress such as post-traumatic stress disorder (PTSD) (17–19). Among Chinese physicians, coping styles appear to mediate 23–30% of overall psychological distress and its three dimensions (depression, anxiety, reduced self-affirmation) (20). Similarly, negative coping among front-line nurses positively correlates with psychological distress during the COVID-19 epidemic (21). Nevertheless, another study found that negative coping styles may have beneficial effects on relieving stress and temporarily coping with setbacks, suggesting that the difference between the two coping styles may be quantitative (22). It indicates the need to investigate whether these coping styles increase or reduce psychological distress among medical staff and medical students during the COVID-19 epidemic.

The definition of social support is a series of support measures accessible to an individual through their social relationships with other individuals, groups, and the larger community. Social support can be divided into three components: subjective support, objective support, and the utilization of support (23). Social support can influence mental and physical health through two possible mechanisms. One is through main effects: social support is salutary for all individuals independent of the extent of stress that they are currently facing. The other mechanism is a stress-buffering model, in which the social support of others may have an ameliorating effect on life stressors, particularly for individuals under greater stress (24). Effective social support can relieve negative emotions caused by stressors as well as improve self-efficacy, which can increase confidence and courage in fighting against crises such as the COVID-19 epidemic (23). Among Chinese medical workers, lack of support from society and patients was identified as an important factor in the workers' psychological burden (25). However, social support is not always beneficial, as one study indicated that Asians are more likely to benefit from implicit social support (social networking), whereas, Caucasians are more likely to benefit from explicit social support (event-specific advice) (26). The potentially complex effect of social support on psychological distress among medical staff and medical students during the COVID-19 epidemic needs to be investigated.

In the early stage of the COVID-19 epidemic, when little was known about the virus and the disease, the individuals may have suffered psychological stress about becoming infected or spreading the virus to their families, friends, or colleagues (27). Perceived psychological stress may increase risk of mental conditions such as depression, anxiety and PTSD (28, 29). Excessive levels of stress can also affect the work environment and produce long-term psychological consequences, especially during an emergency (30). Therefore, studies of people's coping styles, social support and perceived stress during the present epidemic may help guide psychological screening and intervention.

Despite widespread calls for such research, few epidemiological studies have examined psychological distress among medical staff and students, which might serve as the basis for strategies against current and future mental health challenges. The present study aimed to investigate the psychological status and analyzed risk and protective factors of psychological distress among medical staff and medical students in the early stages of the COVID-19 epidemic. We hypothesized that an active coping style and social support were protective factors against psychological distress. We further hypothesized that perceived stress was risk factor against psychological distress among medical staff and medical students. The goal is to provide a scientific basis for psychological interventions and for targeted training programs to strengthen mental health status when facing the epidemic.

## Methods

Medical staff and medical students in China were invited by snowball sampling to participate in this study. All invitees completed the questionnaire online using Questionnaire Star (www.wjx.cn). The initial set of invitees (10 medical staff and 10 medical students) was chosen to ensure broad representation of sex, age, education level, academic or medical specialty, medical or academic institution, and city. Then the questionnaire was forwarded by this set of invitees to 10 colleagues and 10 classmates whom they considered suitable for the survey, and this second set forwarded the questionnaire in the same way, and so on (31).

Inclusion criteria for medical staff were: (1) current engagement in clinical work, (2) possession of a valid medical license, and (3) written informed consent. Inclusion criteria for medical students were: (1) current enrollment in a university or medical institution at any educational level, and (2) written informed consent. Respondents would be excluded if they reported ever having been diagnosed with any disorder listed in the Diagnostic and Statistical Manual of Mental Disorders (4th edition).

Given our desire to assess ~20 factors that might influence psychological distress in our sample, we aimed to recruit at least 10 times as many respondents in order to ensure adequate statistical power (32). We increased this number by 20% to allow for drop-outs, giving a minimal sample size of 220.

### Data Collection

A cross-sectional, Internet-based survey was conducted during February 2–14, 2020. The study was approved by the Ethics Committee of West China Hospital, Sichuan University (No. 2020–178). The complete description of this survey and informed consent form were set prior to questionnaires. After the participants chose “Yes,” the data collection can be continued. Surveys were prepared and administered using Questionnaire Star.

The following validated surveys were administered to all subjects. In addition, they filled out a custom-made questionnaire, designed based on the literature and expert consultation, that collected data on demographics (gender, age, education state, marriage status), place of residence, quality of family relationships, suspected infection of respondents, suspected infection of their family members, hours per day spent watching media coverage of the epidemic, history of visiting Wuhan or contacting with people from Wuhan in recent month and frequency of epidemic-related dreams.

#### Psychological Distress Assessment

The 6-item Kessler Psychological Distress Scale (K6) was used to assess the psychological distress of respondents. It asks about six psychological symptoms during the previous 30 days, including feeling “nervous,” “hopeless,” “restless or fidgety,” “depressed,” “everything is an effort,” and “worthless” (33, 34). Responses on a 5-point Likert scale were scored with “0” (none of the time), “1” (seldom), “2” (some of the time), “3” (most of the time), or “4” (all the time). The total score ranges from 0 to 24 (35). Participants in the present study were categorized as being at low risk of psychological distress (total score of 0–12) or high risk of psychological distress (total score of 13 or more) (36). The scale has proven to show cross-cultural reliability and validity (37, 38). The Chinese version of the K6 has shown moderate to high reliability and validity, with the test-retest reliability was 0.79, Cronbach's alpha was 0.84, split-half coefficient was 0.84, and the correlation between K6 and K10 was 0.961 (39–41).

#### Social Support Assessment

Social support was assessed using the Social Support Rating Scale (SSRS) (42), which consists of 10 items. The scale includes three dimensions: objective support, subjective support and availability of support. The total score is the sum of the scores on each dimension; higher scores reflect more social support. The scale has shown high validity and reliability among Chinese, with a Cronbach's alpha of 0.949 (43).

#### Perceived Stress Assessment

Perceived stress among medical staff and medical students was assessed using the Perceived Stress Scale (44), which measures extent of self-aware stress and the belief that one's life has been overloaded, unpredictable, or uncontrollable during the previous 30 days. The survey includes two dimensions of loss of control and tension, and the 10 items are answered on a 5-point Likert scale. The total score from 0 to 40 is the sum of the scores on the two dimensions; a higher score indicates greater mental stress. The scale has shown high validity and reliability among Chinese (45), with a Cronbach's alpha of 0.82 (46).

#### Coping Style Assessment

Coping style was measured using the Chinese version of the Simplified Coping Style Questionnaire (SCSQ) (22). The 20-item scale consists of two dimensions, positive and negative coping. The first 12 items cover positive coping, and the latter 8 items cover negative coping. The score is based on a 4-point Likert scale (0 = never, 1 = occasionally, 2 = often, 3 = always), with higher scores representing greater positive or negative coping. In the present study, we determined each respondent's coping style based on the difference between the Z-converted standard score for positive coping and the Z-converted standard score for negative coping. If the difference was higher than 0, we considered that the respondent generally adopted a positive coping strategy; otherwise, we considered that the respondent tended to show a negative coping style (47). The scale has shown high reliability and validity among Chinese, with Cronbach's alpha of 0.916 for positive coping and 0.808 for negative coping (22).

### Statistical Analysis

All data were analyzed using SPSS 23.0 (IBM, Chicago, IL, USA). Categorical data were reported as frequencies; continuous data, as mean values. Differences in psychological distress (K6 score) among individuals with different categorical data were assessed for significance using an independent two-samples *t*-test and analysis of variance, while differences in K6 score among individuals with different continuous data were assessed using linear correlation analysis. Stepwise multiple linear regression was performed to identify correlations of psychological distress with demographic characteristics, epidemic-related variables, social support, perceived stress and coping style. Differences associated with *P* < 0.05 were considered statistically significant. All statistical tests were two-tailed.

### Quality Control

The same IP address could be used only once to complete the questionnaire. The survey did not collect any personal information such as names, in order to ensure anonymity and honest responses.

## Results

### Sample Characteristics

A total of 331 medical staff and 249 medical students began completing the surveys. After excluding 27 medical staff and 25 medical students who did not complete them, 304 (91.84%) medical staff and 224 (89.96%) students were included in the final analysis.

Among all medical staff, 210 (69.08%) were women and 94 (30.92%) were men. Ages ranged from 21 to 69 years (mean, 37.15; SD, 9.75), and more than half (74.34%) were unmarried. Among all staff, suspected infection of respondents and their family members were 9.54 and 4.93%, respectively. Fifty-six (18.42%) had a history of visiting Wuhan or being in contact with people from Wuhan in recent months, 9.87% lived in Hubei province, 0.66% reported poor family relationships, 15.79% reported frequent epidemic-related dreams, and 13 (4.28%) spent just a few hours per day watching media coverage of the epidemic.

Among all medical students, 134 (66.67%) were women. Ages ranged from 18 to 32 years (mean, 20.34; SD, 2.41), 95.54% reported good family relationship, suspected infection of respondents and their family members were 64.29% for both. and 148 (66.07%) had a history of visiting Wuhan or being in contact with people from Wuhan in recent months, while 27.86% lived in Hubei province, 144 (64.29%) had frequent epidemic-related dreams, and 7 (3.10%) spent just a few hours each day watching media coverage of the epidemic (Table 1).

**Table 1:** Univariate analysis of factors associated with psychological distress among medical staff and medical students.

**Characteristics**	**Psychological distress of medical staff**	***t/F***	***P-*value**	**Psychological distress of medical students**	***t/F***	***P*-value**
**Gender**						
Male	94 (30.92%)	−0.007	0.994	70 (31.25%)	1.737	0.084
Female	210 (69.08%)			154 (68.75%)		
**Education state**						
Under bachelor's degree	51 (16.77%)	0.783	0.458	43 (19.19%)	14.048	<0.001^**^
Bachelor's degree	187 (61.51%)			161 (71.88%)		
Graduate degree	66 (21.72%)			20 (8.93%)		
**Marriage**						
Yes	78 (25.66%)	2.935	0.004^**^	7 (3.13%)	−0.292	0.771
No	226 (74.34%)			217 (96.87%)		
**Place of residence**						
Non-Hubei province	274 (90.13%)	−3.320	0.001^**^	167 (74.55%)	−6.251	<0.001^**^
Hubei province	30 (9.87%)			57 (25.45%)		
**Family relationship**						
Good	281 (92.43%)	2.817	0.039^*^	214 (95.54%)	19.216	<0.001^**^
General	21 (6.91%)			10 (4.46%)		
Bad	2 (0.66%)			0 (0.00%)		
**Suspected infection of the respondent**						
Yes	29 (9.54%)	−4.617	<0.001^**^	144 (64.29%)	−54.476	<0.001^**^
No	275 (90.46%)			80 (35.71%)		
**Suspected infection of their family members**						
Yes	15 (4.93%)	−6.708	<0.001^**^	144 (64.29%)	−54.476	<0.001^**^
No	289 (95.07%)			80 (35.71%)		
**Spent hours watching outbreaks per day**						
Little (<2 h)	13 (4.28%)	22.095	<0.001^**^	7 (3.10%)	158.636	<0.001^**^
Moderate (2–4 h)	101 (33.22%)			45 (20.10%)		
Much (>4 h)	190 (62.50%)			172 (76.80%)		
**History of visiting Wuhan or contacting with people from Wuhan in recent month**						
Yes	56 (18.42%)	1.927	0.055	148 (66.07%)	38.848	<0.001^**^
No	248 (81.58%)			76 (33.93%)		
**Frequency of epidemic-related dreams**						
Almost never	199 (65.46%)	29.420	<0.001^**^	76 (33.93%)	71.410	<0.001^**^
Sometimes	57 (18.75%)			4 (1.78%)		
Frequent	48 (15.79%)			144 (64.29%)		
**SCSQ**						
Positive coping	279 (91.77%)	−11.904	<0.001^**^	148 (66.07%)	−8.080	<0.001^**^
Negative coping	25 (8.23%)			76 (33.93%)		

* *P < 0.05*;

** *P < 0.01. M, mean; SD, standard deviation; K6, the 6-item Kessler Psychological Distress Scale; SCSQ, Simplified Coping Style Questionnaire*.

#### Psychological Distress, Social Support, Perceived Stress, and Coping Style Among Medical Staff and Medical Students

Medical staff scored a median of 6.77 on the K6, and individuals who scored higher were more likely to develop psychological distress. Average SSRS score was 37.22 ± 11.39, and average PSS score was 18.52 ± 7.54 (Table 2). Most staff (279, 91.77%) showed a positive coping style. Factor values are listed in Table 3. Multivariate analysis identified the following factors as significantly associated with psychological distress among medical staff (Table 4): hours per day spent watching media coverage of the epidemic (β = 1.003, *P* = 0.003), frequent epidemic-related dreams (β = 0.575, *P* = 0.032), social support (β = −0.104, *P* < 0.001), perceived stress (β = 0.285, *P* < 0.001) and coping style (β = 2.520, *P* = 0.004).

**Table 2:** Correlation analysis between factors and psychological distress among medical staff and medical students.

**Characteristics**	**Psychological distress of medical staff**	***r***	***P*-value**	**Psychological distress of medical students**	***r***	***P*-value**
Age	37.15 ± 9.75	−0.156	0.006^**^	20.34 ± 2.41	−0.236	<0.001^**^
SSRS	37.22 ± 11.39	−0.640	<0.001^**^	22.62 ± 11.25	−0.909	<0.001^**^
PSS	18.52 ± 7.54	0.719	<0.001^**^	28.49 ± 11.17	0.946	<0.001^**^

** *P < 0.01. SSRS, Social Support Rating Scale; PSS, Perceived Stress Scale*.

**Table 3:** Variables assessed in the analysis of risk factors for psychological distress among medical staff and medical students.

**Variable**	**Value**
Age	Original value
Gender	0 = male, 1 = female
Education state	0 = under bachelor, 1 = bachelor, 2 = graduate
Marriage	0 = No, 1 = Yes
Family relationship	0 = Good, 1 = Average, 2 = Poor
Spent hours watching outbreaks per day	0 = Little, 1 = Moderate, 2 = Much
History of visiting Wuhan or contacting with people from Wuhan in recent month	0 = No, 1 = Yes
Frequency of recent epidemic-related dreams	0 = Almost never, 1 = Sometimes, 2 = Frequent
SSRS	Original value
PSS	Original value
SCSQ	0 = Positive, 1 = Negative

*SSRS, Social Support Rating Scale; PSS, Perceived Stress Scale; SCSQ, Simplified Coping Style Questionnaire*.

**Table 4:** Analysis of independent risk factors for psychological distress among medical staff.

**Factors**	**Unstandardized coefficients**		**Standardized coefficients beta**	***t***	***P*-value**	**95%CI**
	**β**	**SE**				
Constant	2.703	1.415	-	1.910	0.057	−0.082–5.487
Spent hours watching outbreaks per day	1.003	0.339	0.114	2.962	0.003^**^	0.337–1.670
Frequency of recent epidemic-related dreams	0.575	0.267	0.086	2.157	0.032^*^	0.050–1.100
SSRS	−0.104	0.022	−0.234	−4.708	<0.001^**^	−0.147 to −0.060
PSS	0.285	0.035	0.426	8.040	<0.001^**^	0.215–0.355
SCSQ	2.520	0.865	0.138	2.913	0.004^**^	0.818–4.223

* *P < 0.05*;

** *P < 0.01. SSRS, Social Support Rating Scale; PSS, Perceived Stress Scale; SCSQ, Simplified Coping Style Questionnaire*.

Medical students scored a mean of 15.48 on the K6; their average SSRS score was 22.62 ± 11.25, and their average PSS score was 28.49 ± 11.17 (Table 2). A small majority (148, 66.07%) showed a positive coping style. Multivariate analysis identified the following factors as significantly associated with psychological distress among students (Table 5): hours per day spent watching media coverage of the epidemic (β = 1.679, *P* < 0.001), frequent epidemic-related dreams (β = 3.745, *P* < 0.001), social support (β = −0.135, *P* < 0.001), and perceived stress (β = 0.256, *P* < 0.001).

**Table 5:** Analysis of independent risk factors for psychological distress among medical students.

**Factors**	**Unstandardized coefficients**		**Standardized coefficients beta**	***t***	***P*-value**	**95%CI**
	**β**	**SE**				
Constant	−0.343	1.736	-	−0.198	0.843	−3.764 to 3.077
Spent hours watching outbreaks per day	1.679	0.436	0.098	3.848	<0.001^**^	0.819–2.539
Frequency of recent epidemic-related dreams	3.745	0.564	0.409	6.638	<0.001^**^	2.633–4.857
SSRS	−0.135	−0.175	−0.175	−3.792	<0.001^**^	−0.204 to −0.065
PSS	0.256	0.330	0.330	5.955	<0.001^**^	0.171–0.341

** *P < 0.01. SSRS, Social Support Rating Scale; PSS, Perceived Stress Scale*.

## Discussion

The current study assessed the prevalence of psychological distress among Chinese medical workers and medical students during the early stages of the COVID-19 epidemic, and it explored potential correlations of that distress with social support, perceived stress, and coping style. Similar to previous bio-disasters including SARS, Ebola, H1N1 influenza and MERS epidemics, the COVID-19 epidemic appears to have strongly adverse psychological effects on medical staff, such as depression, anxiety and insomnia (48).

### Psychological Distress Among Medical Staff and Medical Students

The present results about psychological distress among medical staff are consistent with a previous study among Chinese medical staff (48). The study among healthcare workers in Ireland reflected that 42.6% for depression and 45.1% for both anxiety and stress (49). Also, there were study indicated that during the outbreak, the prevalence of depressive was in 27.5–50.7%, insomnia was in 34–36.1%, and severe anxiety in 45% among Italian healthcare workers (50). However, a study on Singapore healthcare workers revealed a lower prevalence with a proportion of 5.3 on depression and 8.7 on anxiety, 3.8% of them screened for moderate to severe levels of psychological distress during the COVID-19 epidemic (51). The discrepancy of psychological impact of COVID-19 on healthcare workers may reflect the different epidemic situation in different counties in the early stages of COVID-19 outbreak.

The present study further showed that a substantial proportion of medical students also experienced psychological distress during the initial stages of the COVID-19 epidemic. Previous studies found prevalence of anxiety to be 24.9% and prevalence of depression to be 40.5% among medical students during the COVID-19 epidemic (52, 53). These prevalence are much higher than those in the general Chinese population (54). A survey on Australian medical students revealed a mean K10 score of 20.6 indicating moderate psychological distress (55). As reported in a study on Iranian medical students, the prevalence of anxiety was 38.1% and depression was 27.6% (56). Also, a previous study on home-quarantined Bangladeshi students reflected that, 28.5% of them had stress, 33.3% had anxiety and 46.92% had depression from mild to extremely severe (57). These higher prevalence may reflect that, because schools have been closed, medical students tend to receive COVID-19 information more from social media rather than from scientific sources (58), which may lead to inaccurate assessment of the epidemic situation, leading in turn to excessive stress and concern that compromises their ability to gain professional knowledge in school (12).

Our results are consistent with the idea that the COVID-19 epidemic has placed a substantial burden on the mental health of medical staff and medical students in China. Therefore, psychological interventions should be provided urgently not only for medical staff but also for medical students, who are the reserve forces for medical staff. Such interventions should aim to enhance mental health during the COVID-19 epidemic.

### Factors Influencing Psychological Distress Among Medical Staff and Medical Students

Multilinear regression identified social support, perceived stress, hours per day spent watching media coverage of the epidemic, and frequency of recent epidemic-related dreams as factors significantly influencing psychological distress among medical staff and medical students. Coping style was identified as another influencing factor among medical staff.

#### Social Support

Social support was identified as a factor influencing psychological distress in medical staff and medical students. Individuals who reported more social support were less likely to develop psychological distress. This is consistent with previous studies of Chinese medical workers (42, 59). Several studies have emphasized the role of social support in protecting mental health of various populations, including medical students (52, 60, 61). For example, inadequate support from family and friends has been associated with significantly greater risk of depression among US medical students (61), and a study of Australian medical students found similar results (62). Social support from friends or family can help medical staff reduce anxiety and stress, by reducing the perceived threat and inappropriate behavior that can result from stress events (63, 64). Social support can also improve self-efficacy, leading to more understanding, encouragement, courage, and a sense of professional achievement, resulting in increased confidence and optimism, which improves positive coping when facing stress (65, 66).

Psychological resilience may partially mediate the effects of social support on mental health, as suggested by a study of Chinese health care workers during the peak of the COVID-19 epidemic (59). Resilience has been positively associated with social support during the aftermath of major disasters: a study of adolescent survivors of the Wenchuan earthquake found that resilience can help protect individuals against mental illness (67, 68). This positive correlation has been observed across different populations faced with different disasters (69–72). Therefore, institutions should pay more attention to providing their staff with support that complements the social support they receive from families and healthcare authorities. More importantly, medical schools can embed training in emotional resilience into the curriculum in order to reduce psychological distress among medical students in daily life and emergency events (62).

#### Perceived Stress

In the present study, a higher level of perceived stress among medical staff or medical students was associated with greater likelihood of developing psychological distress. A study of medical staff in Guangdong, China found that individuals with moderate-to-severe anxiety or depressive symptoms were more likely to perceive higher stress (73), and perceived stress has been shown to predict anxiety among the general Chinese population during COVID-19 (46). A study of women in the US found that stressful life events were significantly associated with depression (74). Our results with medical students are consistent with a previous study suggesting that anxiety and depression among medical students are significantly related to their stress (75). Perceived stress reflects one's psychological experience after the self-interpretation of stressful event (76). A higher score is associated with higher risk of developing mental illness. Psychological stress may weaken immunity, resulting in a higher risk of infection and mental illness (77, 78).

In addition to the social support mentioned above, resilience can also alleviate the adverse effects of stress on medical workers and students (79, 80). For example, resilience negatively correlates with perceived stress among Chinese medical staff during COVID-19 (81). A study of medical staff during the SARS epidemic found that measures to increase resilience reduced perceived stress among medical staff (82). Another study found that resilience among medical students can protect them from stress (83). This protective role of resilience may help guide the design of measures to alleviate the stress of medical workers and medical students during the COVID-19 epidemic as well as during normal professional and personal life (84).

### Hours per Day Spent Watching Media Coverage of the Epidemic

Medical staff and medical students in our study who spent more time daily watching media coverage of the epidemic were more likely to develop psychological distress. Similar results were reported in a study of the general Chinese population (85). During the early stage of the epidemic, media reports may have caused intense worry and panic by highlighting the government's efforts to fight against the outbreak, protective interventions, numbers of suspected infections and confirmed cases every day, while also highlighting the lack of effective treatments (85). At the same time, medical staff are concerned about their own health and about the risk of transmitting infection to their families. The more time they spend on searching for information about the epidemic, the more anxiety, stress or fear they report (86–88).

Medical students, in contrast, have tended to depend more on social media rather than scientific sources to obtain information about the epidemic and prevention measures, which may lead to inaccurate assessment of the epidemic situation (58). The frequent mention of the outbreak in the media and excessive attention paid to it may also aggravate their concerns and fears, compromising their ability to learn professionally about it (12, 89). Our results support the idea that medical students' self-confidence in coping with COVID-19 can be increased by giving priority to traditional national media directly connected to trustworthy medical decision-makers (90).

### Frequency of Epidemic-Related Dreams

Frequency of epidemic-related dreams was significantly associated with psychological distress among medical staff and medical students in our study. Similar results have been reported in a study of the general Chinese population (54). Sleep problems, especially dreams in which the content relates directly to the traumatic event, are core symptoms of PTSD (91). This suggests that Chinese medical staff and medical students may have experienced PTSD symptoms in the early stages of the COVID-19 epidemic.

### Coping Style

Multivariate analysis also showed that coping style was an important factor influencing psychological distress among the medical staff in our study. Medical staff with a positive coping style were less likely to report psychological distress. Several studies have linked negative coping style with subsequent mental illness, and positive coping style with better mental health (20, 92, 93). Indeed, these results have been reported for the general Chinese population during COVID-19 (54), as well as for Romanian healthcare workers (94). Therefore, appropriate psychological interventions should be urgently provided to medical workers with negative coping styles during COVID-19.

Among medical students in our study, coping style did not emerge from multivariate analysis as significantly associated with psychological distress, although it was significant in single-factor analysis (see Table 1). These results suggest that coping style may not be a major determinant of psychological distress among medical students. It is also possible that our sample was too small to detect an association.

### Limitations

This study was conducted during the early stages of the COVID-19 epidemic, only a few days after the entire city of Wuhan was placed under quarantine. While it may give a reasonably accurate view of the situation early in the epidemic, our results should be interpreted with caution given several limitations. One is the on-line format, necessary in large part because of the inability for us to interact face-to-face with potential respondents. So it is unclear whether our results can be generalized to people without Internet access. Secondly, the snowball sampling method may cause selection bias which may reduce the generalizability of our study. Thirdly, we did not assess whether and how respondents were engaging in prevention, as preventive behaviors can also play a role in mediating stress levels (95). Fourthly, the influence factors related to COVID-19 epidemic would change and the starting situations were different in different counties. However, our study may benefit to develop targeted training programs to strengthen mental health status of medical staffs and students when facing the similar infectious disease epidemic in the future in different countries. Finally, our cross-sectional study could not capture changes in psychological distress or identify its predictors during the course of the COVID-19 epidemic. Therefore, future studies would be to convey a follow-up for the current situation and engage in a more consistent analysis about the long-term psychological effects of the COVID-19 epidemic among medical staff and medical students. Such work should also further explore the ability of social support and coping strategies to mediate the effects of the COVID-19 epidemic on psychological distress and mental health more generally.

The COVID-19 epidemic in China has substantially affected the mental health of medical staff and medical students. Urgent mental health interventions should be implemented in a timely manner in order to prevent psychological distress and promote recovery. Our study has associated higher social support, lower perceived stress and less time spent daily watching media coverage of the epidemic with lower psychological distress among medical staff and medical students in the early stages of the COVID-19 epidemic. Medical staff with a positive coping style may also have lower psychological distress. Our results have several practical implications. Medical staff and medical students may benefit from being taught positive coping strategies and being encouraged to seek and maintain social support. Such interventions may help protect their mental health not only during the current COVID-19 epidemic but also during future public health emergencies. Most importantly, they should regularly receive comprehensive, systematic training in order to be more resilient to the daily pressures of their work. To benefit medical students, who are the reserve forces supporting medical staff, medical schools should use social media more frequently to disseminate knowledge and develop training plans (53). Medical schools should also consider adding training in mental resilience for emergency events into their curricula (61).

## Data Availability Statement

The raw data supporting the conclusions of this article will be made available by the corresponding authors on reasonable request.

## Ethics Statement

The studies involving human participants were reviewed and approved by the Ethics Committee of West China Hospital, Sichuan University and run in accordance with the 1964 Helsinki declaration and its later amendments or comparable ethical standards. Written informed consent to participate in this study was obtained from all individual participants included in this study through the Web-based surveys.

## Author Contributions

ZheL and XY developed concept, study design, and wrote the original paper. MZ, ZhiL, and WX collected and analyzed the data. SW and ZX made critical revision of the manuscript for important intellectual content. All authors read and approved the final manuscript.

## Conflict of Interest

The authors declare that the research was conducted in the absence of any commercial or financial relationships that could be construed as a potential conflict of interest.
